# Neuroprotection by Combined Administration with Maslinic Acid, a Natural Product from *Olea europaea*, and MK-801 in the Cerebral Ischemia Model

**DOI:** 10.3390/molecules21081093

**Published:** 2016-08-19

**Authors:** Yisong Qian, Xuzhen Tang, Teng Guan, Yunman Li, Hongbin Sun

**Affiliations:** 1 Institute of Translational Medicine, Nanchang University, 1299 Xuefu Avenue, Nanchang 330001, China; 2 Department of Physiology, China Pharmaceutical University, 24 Tongjiaxiang Street, Nanjing 210009, China; txzgrace@gmail.com; 3 Department of Human Anatomy and Cell Science, University of Manitoba, 745 Bannatyne Avenue, Winnipeg, MB R3E 0J9, Canada; guan.teng@gmail.com; 4 Center for Drug Discovery, China Pharmaceutical University, 24 Tongjiaxiang Street, Nanjing 210009, China; hbsun2000@yahoo.com

**Keywords:** cerebral ischemia, maslinic acid, MK-801, GLT-1

## Abstract

Glutamate-mediated excitotoxicity is a major cause of ischemic brain damage. MK-801 confers neuroprotection by attenuating the activation of the *N*-methyl-d-aspartate (NMDA) receptor, but it failed in clinical use due to the short therapeutic window. Here we aim to investigate the effects of maslinic acid, a natural product from *Olea europaea*, on the therapeutic time window and dose range for the neuroprotection of MK-801. Rats were administered with maslinic acid intracerebroventricularly and cerebral ischemia was induced by middle cerebral artery occlusion (MCAO) followed by reperfusion. MK-801 was administered at 1 h, 2 h, 3 h and 4 h after ischemia, respectively. The cerebral infarct volume was determined by 2,3,5-Triphenyltetrazolium chloride (TTC) staining, neuronal damage was assessed by Haematoxylin Eosin (H&E) staining, and the expression of glial glutamate transporters and glial fibrillary acidic protein (GFAP) was evaluated by immunohistochemistry and Western blot post-ischemia. Results showed that the presence of maslinic acid extended the therapeutic time window for MK-801 from 1 h to 3 h. Co-treatment of maslinic acid and MK-801 at a subthreshold dosage obviously induced neuroprotection after ischemia. The combination of these two compounds improved the outcome in ischemic rats. Moreover, maslinic acid treatment promoted the expression of GLT-1 and GFAP post-ischemia. These data suggest that the synergistic effect of maslinic acid on neurological protection might be associated with the improvement of glial function, especially with the increased expression of GLT-1. The combination therapy of maslinic acid and MK-801 may prove to be a potential strategy for treating acute ischemic stroke.

## 1. Introduction

Stroke represents the leading cause of death and permanent disability for old people. Up to now, no single pharmacological agents have shown any neurological improvement for patients with acute ischemic stroke except thrombolytic drugs, despite promising preclinical data [1,2]. Several key factors have been implicated in ischemic cell death, including excitotoxicity, oxidative and nitrosative stress, and inflammation [3]. Excessive release of glutamate and *N*-methyl-d-aspartate (NMDA) receptor-related excitotoxicity has been regarded as major causes of brain tissue damage [1]. One approach to addressing these problems is to lower the concentration of extracellular glutamate via blocking its release or increasing the intake [4,5]. Another way is to attenuate the activation of the NMDA receptor with NMDA receptor antagonists.

**Figure 1:**
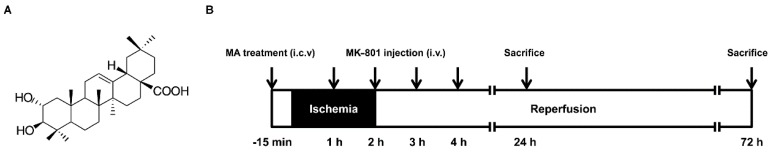
(**A**) Chemical structure of maslinic acid; (**B**) Schematic representation showing the timeline of the experimental protocols. Maslinic acid was administered intracerebroventricularly (i.c.v.) 15 min before MCAO. MK-801 was administered at the indicated time points after the induction of ischemia through intravenous injection (i.v.).

MK-801, an NMDA receptor antagonist, affords reliable protection against ischemic injury in experimental focal stroke [6]. However, clinical trials using MK-801 as a protective agent against acute ischemic stroke have failed due to its short therapeutic window. MK-801 reduced the cortical infarct volume when given just prior to ischemia, but had no neuroprotective activity when given 30 min after the onset of ischemia [7], suggesting that MK-801 plays only a transient role in reversible focal ischemia. Moreover, a high dosage of MK-801 may cause side effects including behavioral toxicity and psychotomimetic effects [8], which also restrict its clinical use. There is accumulating evidence that a combination treatment with MK-801 and other agents synergistically reduced infarct volume and improved neurological deficits in experimental stroke. These effects may be partially attributed to other pathways and non-excitotoxic processes activated by the combination strategy [9,10]. These studies suggested a possibility for the new use of NMDA receptor blockers in the treatment of stroke. Maslinic acid (2-α,3-β-dihydroxyolean-12-en-28-oic acid) is an oleanane-type triterpenoid compound abundantly expressed in the wax skin of *Olea europaea*, a species of small tree in the family Oleaceae (Figure 1A) [11]. It was previously demonstrated as a potent inhibitor of astrocytic glycogen phosphorylase with low toxicity [12,13], and has been applied in pathological processes including hypoglycemia and diabetes [14,15] due to its glucose-lowering effects by interfering with abnormal glycogen metabolism. We have reported that maslinic acid regulated the extracellular glutamate concentration by increasing the expression of astrocytic glutamate transporters both in vivo and in vitro, thus providing neuroprotection against excitotoxic injury [15,16]. In this regard, we hypothized that the mechanism of neuroprotection by maslinic acid may be associated with improved glutamate metabolism. The main objective of the present study is to determine the time window for neuroprotection of the combination treatment with MK-801 and maslinic acid in a rat model of cerebral ischemia. We also investigated whether the combination protocol could allow a lowered dosage of MK-801 to a yield positive neurologic outcome. Furthermore, since glutamate transporters have been shown to be involved in the mechanism of maslinic acid-mediated neuroprotection, the expression of two glutamate transporters, GLAST and GLT-1, was also examined after ischemic stroke.

## 2. Results

### 2.1. Physiological Variables

Physiological variables, including arterial blood pressure, blood gases and rectal temperatures, were not significantly different between any of the experimental groups before, during, or after middle cerebral artery occlusion (MCAO). The laser Doppler flowmetry signal showed no significant differences among groups before surgery. Compared with the sham-operated group, regional cerebral blood blow (rCBF) dropped to <25% of the baseline at 1 h after MCAO. After 1 h of reperfusion, the rCBF value showed a slight increase compared with the sham-operated group, but did not differ significantly in each group subjected to MCAO (data not shown).

### 2.2. Neuroprotective Effects of Maslinic Acid and MK-801 on Focal Cerebral Ischemia in Rats

**Figure 2:**
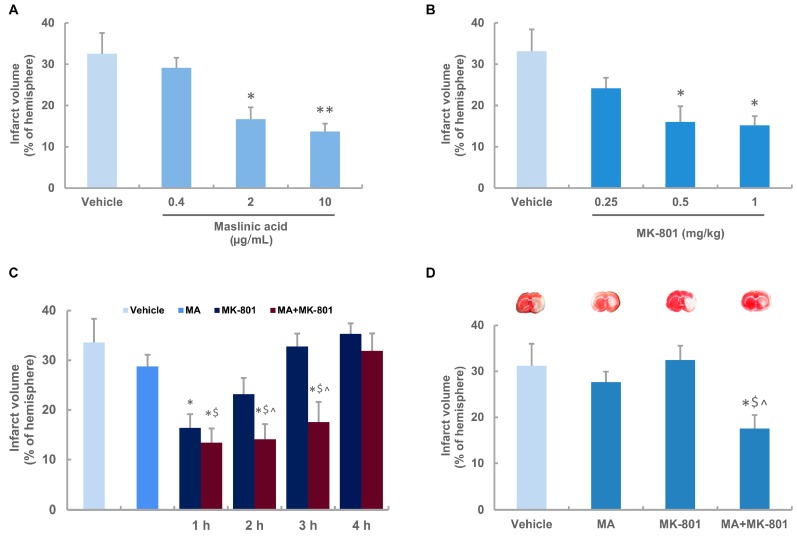
Neuroprotective effects of maslinic acid and MK-801 on focal cerebral ischemia in rats subjected to 2 h of ischemia and 22 h of reperfusion. (**A**) A dose-response analysis of maslinic acid on infarct volume; (**B**) A dose-response analysis of MK-801 on infarct volume. MK-801 was given at 1 h after MCAO; (**C**) Maslinic acid extends the time window for the efficiency of MK-801 in ischemic rats. Maslinic acid (0.4 μg/mL) was administered 15 min before MCAO. MK-801 (0.5 mg/kg) was given at the indicated time points after MCAO alone or combined with maslinic acid; (**D**) Maslinic acid induces a lower dose of MK-801 to exert neuroprotection in ischemic rats. Rats were treated with vehicle, maslinic acid (0.4 μg/mL, 15 min before MCAO), MK-801 (0.25 mg/kg, 1 h after MCAO) or MK-801 combined with maslinic acid, respectively. Each column represented as mean ± SEM (*n* = 8 for each group). * *p* < 0.05, ** *p* < 0.01 versus the vehicle-treated group; $ *p* < 0.05 versus maslinic acid-treated group; ^ *p* < 0.05 versus MK-801-treated group.

To investigate the dose response and time response of maslinic acid and MK-801 on focal cerebral ischemia, maslinic acid (0.4 μg/mL, 2 μg/mL and 10 μg/mL) or artificial cerebrospinal fluid (aCSF) was administered intracerebroventricularly 15 min before ischemia induction, and MK-801 was administered by intravenous injection (i.v.) at different time points after ischemia onset. Rats were sacrificed at 22 h after reperfusion for the assessment of infarct volumes (Figure 1B). Compared with the vehicle group, a significant reduction in infarct volume (by 48% and 58%) was observed in maslinic acid-treated rats at the concentrations of 2 μg/mL and 10 μg/mL, while treatment with maslinic acid at 0.4 μg/mL was not effective in reducing infarct volume (Figure 2A). Considering the compound per se shows potent efficacy in ischemic injury, we applied 0.4 μg/mL of maslinic acid (subthreshold dosage) in the combination study to certify the synergistic effect.

MK-801 (0.25 mg/kg, 0.5 mg/kg and 1 mg/kg) or saline was injected i.v. 1 h after ischemia. Compared with the saline group, MK-801 (0.5 mg/kg and 1 mg/kg) treatment effectively prevented brain damage, with a reduction in infarct volume by 48% and 53%, respectively. MK-801 at 0.25 mg/kg had no effect on infarct volume induced by MCAO (Figure 2B). Therefore, 0.5 mg/kg of MK-801 as an effective dosage was used in the time window study, and MK-801 at 0.25 mg/kg as a subthreshold dosage was applied in the combination study.

### 2.3. Maslinic Acid Extends the Time Window and Decreases the Dosage of MK-801 in Ischemic Rats

To investigate if MK-801 could provide additional neuroprotection or reveal a prolonged time window in the presence of maslinic acid, MK-801 at 0.5 mg/kg was administered at 1 h, 2 h, 3 h or 4 h after ischemia, respectively, with or without maslinic acid treatment at 15 min before MCAO. Results showed that a single injection of MK-801 at 1 h after ischemia could obviously reduce the infarct volume (*p* < 0.05 compared with the vehicle group), but did not show significant protection at 2 h, 3 h and 4 h time points. Maslinic acid (0.4 μg/mL) alone was not able to produce neuroprotection. However, MK-801 effectively prevented brain damage in the presence of maslinic acid within 3 h after MCAO (*p* < 0.05 compared with the vehicle group). In addition, the combination group at 1 h, 2 h and 3 h time points showed a significant reduction in infarct volume compared with the maslinic acid-treated group (*p* < 0.05). The combination treatment exerted neuroprotection at 2 h and 3 h following ischemia, when a single injection of MK-801 was ineffective (*p* < 0.05, Figure 2C). These data indicated that the time window for the efficiency of MK-801 was prolonged from 1 h to 3 h when combined with the subthreshold dosage of maslinic acid pretreatment.

We further investigated if both the subthreshold dosages of maslinic acid and MK-801 treatment could induce neuroprotection in ischemic rats. We demonstrated that neither maslinic acid nor MK-801 was neuroprotective at a subthreshold dosage. However, the combination therapy showed synergistic effects on infarct volume compared with the vehicle or the single treatment group, when maslinic acid (0.4 μg/mL) was given 15 min before MCAO followed by MK-801 (0.25 mg/kg) administration 1 h after MCAO (Figure 2D).

### 2.4. MK-801 Combined with Maslinic Acid Improves the Outcome in Rats Subjected to Cerebral Ischemia

**Figure 3:**
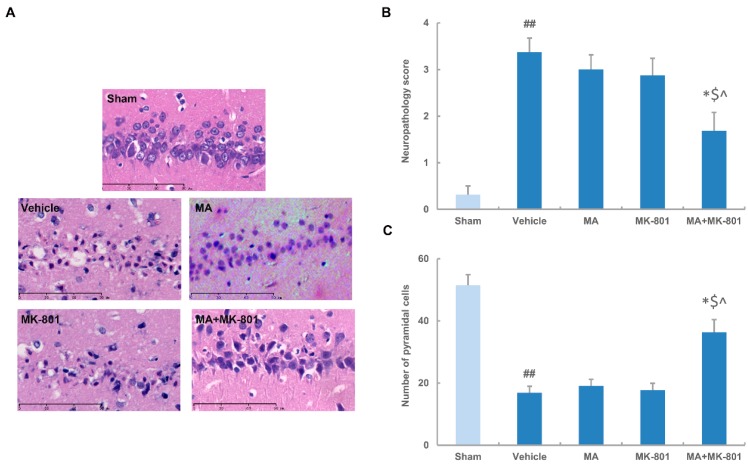
MK-801 combined with maslinic acid improves ischemic outcome in rats. (**A**) Hippocampal neuronal damage in CA1 subregion by H&E staining. Rats were subjected to 2 h of ischemia followed by 70 h of reperfusion. Maslinic acid (0.4 μg/mL) was administered 15 min before MCAO. MK-801 (0.5 mg/kg) was given at 3 h after MCAO; (**B**) Analysis of neuronal damage by neuropathalogical score; (**C**) Number of pyramidal neurons was counted in CA1 subregion. Data were expressed as means ± SEM from three independent animals containing three randomly selected fields. ## *p* < 0.01 versus the sham group; * *p* < 0.05 versus the vehicle group; $ *p* < 0.05 versus maslinic acid-treated group; ^ *p* < 0.05 versus MK-801-treated group. Scale bar: 90 μm.

Rats were sacrificed 70 h after MCAO for histological assay (Figure 1B). In the sham-operated group, nearly all pyramidal neurons in the CA1 region were arranged in order with intact outlines. In the vehicle group, most cells appeared with shrunken, triangulated or pyknotic cell bodies. The cellular inter-space widened and neurons were arranged asymmetrically. Maslinic acid (0.4 μg/mL) pretreatment 15 min before MCAO followed by MK-801 (0.5 mg/kg) injection 3 h post-ischemia significantly improved the outcome while MK-801 or maslinic acid alone did not alter the histological appearance compared with the vehicle group (Figure 3A). The vehicle group demonstrated a high neuropathalogical score, which was effectively improved in the presence of maslinic acid and MK-801. However, maslinic acid or MK-801 alone has few effects on the neuropathological score after ischemic insults (Figure 3B). The number of normal neurons subjected to ischemia decreased to 32.8% compared with the sham group. The single treatment of maslinic acid or MK-801 at a subthreshold dosage did not affect the neuron count in the CA1 subregion, while the combination protocol obviously increased the number of pyramidal cells following ischemia/reperfusion injury (Figure 3C).

### 2.5. Maslinic Acid Up-Regulates the Expression of GLT-1 after Cerebral Ischemia

**Figure 4:**
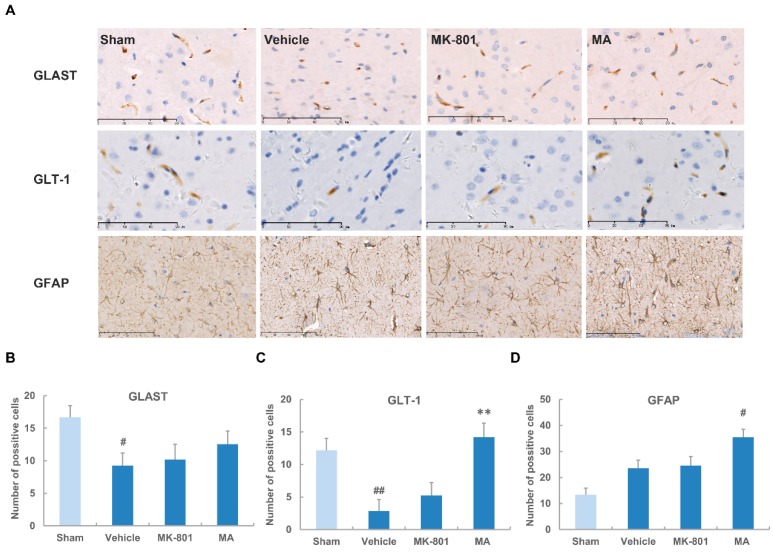
Maslinic acid increases the expression of glial glutamate transporters after cerebral ischemia. (**A**) The expression of GLAST, GLT-1 and GFAP was determined by immunohistochemistry following 2 h of ischemia and 70 h of reperfusion. Maslinic acid (0.4 μg/mL) was administered 15 min before MCAO. MK-801 (0.5 mg/kg) was given at 3 h after MCAO; The expression of (**B**) GLAST; (**C**) GLT-1; and (**D**) GFAP was quantitatively represented as the positive-cell number in one field. Data were expressed as means ± SEM from three independent animals containing three randomly selected fields. # *p* < 0.05, ## *p* < 0.01 versus the sham group; ** *p* < 0.01 versus the vehicle group. Scale bar: 90 μm.

The astrocytic glutamate transporters, GLAST and GLT-1, have been considered to control the extracellular glutamate concentration and play crucial roles in ischemic brain injury. Therefore, the expressions of GLAST and GLT-1 were determined in the ischemic region. After 2 h of MCAO followed by 70 h of reperfusion, GLAST-positive cells were decreased by 44% (*p* < 0.05) compared with the sham-operated group. Neither maslinic acid (0.4 μg/mL) nor MK-801 (0.5 mg/kg) had significant effects on the expression of GLAST (Figure 4A,B). GLT-1–positive cells were reduced by 76% in the vehicle group. Maslinic acid induced a marked increase in GLT-1–positive cells (*p* < 0.01), while no significant difference was observed in the MK-801 group after ischemia compared with the vehicle group (Figure 4A,C).

GFAP is a specific biomarker for astrocytes. Increased expression of GFAP represents astroglial activation during cerebral ischemia. According to our study, the expression of GFAP was up-regulated in the vehicle group, with the increased number of GFAP-positive astrocytes and intensive foot processes, which were not statistically different from the sham-operated group, indicating a slight post-ischemic activation of astrocytes. Pretreatment with maslinic acid further increased the number of GFAP-positive astrocytes while MK-801 showed little influence on GFAP expression (Figure 4A,D).

**Figure 5:**
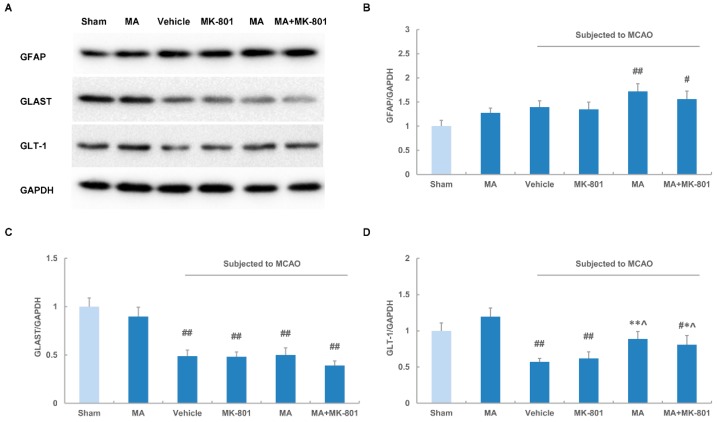
Maslinic acid up-regulates the levels of glial glutamate transporters after cerebral ischemia. (**A**) The immunoblots of GFAP, GLAST, GLT-1 and GAPDH were obtained by Western blot following 2 h of ischemia and 70 h of reperfusion. Maslinic acid (0.4 μg/mL) was administered 15 min before MCAO. MK-801 (0.5 mg/kg) was given at 3 h after MCAO; Quantitative analysis of (**B**) GFAP; (**C**) GLAST; and (**D**) GLT-1 levels normalized to GAPDH. Data were expressed as means ± SEM from three independent animals. ## *p* < 0.01 versus the sham group; * *p* < 0.05, ** *p* < 0.01 versus the vehicle group; ^ *p* < 0.05 versus MK-801-treated group.

We further performed Western blot analysis to observe the effects of maslinic acid and the combination treatment on the expression of GFAP, GLAST and GLT-1. We found that maslinic acid treatment induced a slight increase in GFAP levels in sham rats. MCAO did not alter the expression of GFAP significantly while maslinic acid or maslinic acid plus MK-801 markedly promoted its expression compared with the sham rats. MK-801 alone had no effects on GFAP activation (Figure 5A,B). In a coincidence with the immunohistochemistry results, GLAST expression was decreased after MCAO and maslinic acid or MK-801 did not reverse its down-regulation. In addition, maslinic acid alone had no effects on GLAST levels in sham rats (Figure 5A,C). Maslinic acid treatment increased GLT-1 levels, which was not significantly different from that in sham rats. However, maslinic acid obviously prevented the reduction of GLT-1 following cerebral ischemia. The combination group showed similar effects on GLT-1 expression compared with the single treatment of maslinic acid, while MK-801 alone did not produce a significant increase in GLT-1 levels after MCAO (Figure 5A,C).

## 3. Discussion

The present study demonstrated that the combined treatment with maslinic acid significantly extended the therapeutic time window for MK-801 from 1 h to 3 h. The subthreshold dosage of maslinic acid allowed a much lower dose of MK-801 to produce a significant reduction in infarct volume. In addition, the combination of these two compounds also improved the outcome in ischemic rats. These results indicated that the combination therapy of maslinic acid and MK-801 may be a potential strategy for treating acute ischemic stroke. 

Excessive stimulation of neuronal glutamate receptors contributes mainly to glutamate-mediated excitotoxicity after ischemia. Activation of glutamate receptors has been shown to increase energy consumption as well as free radical production, which in turn impairs glutamate uptake and causes further elevation of extracellular glutamate [17]. Excitotoxic cell death appears to be mediated by different types of glutamate receptors, among which the NMDA subtypes are particularly involved [18]. Apart from the excess of glutamate release, a deficiency in glutamate clearance from the synaptic cleft also results in the accumulation of extracellular glutamate, which further promotes rapid activation of NMDA receptors and proceeds peripherally from the ischemic core into the penumbral region [4]. MK-801, an NMDA receptor antagonist, could effectively avoid excessive activation of glutamate receptors, and thus affords protection against ischemic injury. MK-801 given prior to or just after the onset of focal ischemia could effectively prevent glutamate extension, thus protecting brain cells from injury. However, it is less effective after irreversible activation of the NMDA receptor has appeared [7]. This might lead to the narrow therapeutic time window of MK-801 in treating acute ischemic stroke.

The intracerebroventricular (i.c.v.) route is recognized as providing a method for bypassing the blood-brain barrier and directly delivering therapeutic drugs to the ischemic regions of the central nervous system (CNS) rapidly after the onset of stroke. Of particular clinical interest, the i.c.v. route can be effectively substituted for intranasal administration [10]. Therefore, we employed i.c.v. administration for the time window study to investigate the synergistic effects of maslinic acid. In agreement with previous findings [15], maslinic acid showed dose-dependent neuroprotection in ischemic rats. Pretreatment with maslinic acid at a subthreshold dosage extended the time window and decreased the effective dosage of MK-801 in experimental stroke, which indicated a significant increase in effectiveness and a decreased risk in psychoactive effects. The combination of these two drugs also showed a marked reduction in CA1 cell damage, rather than failure to protect white matter or oligodendrocytes as previously reported [9]. Taken together, the potent protection and low risk of the combined treatment may be a promising new therapeutic strategy in clinical application.

The clearance of excessive glutamate from the synaptic cleft depends mainly on its transport by high-affinity, Na^+^-dependent carriers (termed GLAST and GLT-1) localized on astrocytes [19]. GLT-1 is the predominant glial glutamate transporter in the hippocampus and is responsible for clearing most of the synaptically released glutamate. Decreased GLT-1 levels after ischemia and during the reperfusion periods could promote neuronal death by slowing the reuptake of released glutamate [20]. We have previously reported that maslinic acid increased the expression of glial glutamate transporters at the protein and mRNA levels in astrocytes in vitro [16]. To explore the mechanisms underlying the synergistic effect of maslinic acid, the role of glial glutamate transporters in ischemic rats was investigated. Not in full agreement with our in vitro study, neither GLAST nor GLT-1 increased significantly in sham animals exposed to maslinic acid. This difference may be due to the duration of drug exposure or other factors influencing the expression of glutamate transporters in the in vivo experiment. Both immunostaining and Western blot analysis showed an obvious reduction in GLT-1 and GLAST protein levels after transient ischemia, which is in accordance with previous studies [21]. Maslinic acid treatment effectively antagonized ischemia-induced down-regulation of GLT-1, thus increasing the capacity for glutamate uptake during ischemia, and led to a likely lower level of extracellular glutamate. As a result, the delayed expansion of excitotoxicity into the cortical penumbral region and the excessive activation of NMDA receptors could be prevented, allowing more regions to be protected by MK-801 administration after ischemia. This may explain the prolonged time window for MK-801 in the presence of maslinic acid.

Astrocyte activation plays a crucial role in maintaining the stability of the internal environment and the survival of neurons in ischemic brain injury [22]. GFAP, the major intermediate filament of astrocytes, is thought to mainly participate in the regulation of neuron construction, amino acid transport and myelinogenesis [23,24]. The up-regulation of GFAP is the hallmark of reactive astrocytes. In our study, maslinic acid slightly elevated GFAP levels after ischemia compared with vehicle treatment, indicating the involvement of maslinic acid in the functional role of reactive astrocytes. Moreover, it has been demonstrated that moderately reactive astrocytes maintain overall glutamate transporter expression and function, which contribute to decreased extracellular glutamate concentrations and prevent excitotoxic cell damage [25]. However, maslinic acid did not affect GFAP activation when it was given to rats not subjected to MCAO, suggesting that maslinic acid had few effects on glial function under normal physiological conditions. Therefore, maslinic acid promoted glutamate clearance probably either by direct up-regulation of GLT-1 or indirect enhancement of astrocyte function.

## 4. Material and Methods

### 4.1. Materials

MK-801 and 2,3,5-triphenyltetrazolium chloride (TTC) were purchased from Sigma (St. Louis, MO, USA). Maslinic acid (96.5 percent purity, No. 0060324) was supplied by Center for Drug Discovery, China Pharmaceutical University. Rabbit anti-GFAP antibody was obtained from DAKO (Glostrup, Denmark), and rabbit anti-GLAST and anti-GLT-1 antibodies were from Santa Cruz Biotechnology (Santa Cruz, CA, USA).

### 4.2. Animal Model

All experiments were approved by the ethics committee of China Pharmaceutical University. Animal experiments were carried out in accordance with the National Institutes of Health Guide for the Care and Use of Laboratory Animals. Adult male Sprague-Dawley rats, 240–260 g in weights, seven to eight weeks old, were provided by the Experimental Animal Center of China Pharmaceutical University. Rats were housed in a temperature-controlled environment (18–22 °C) with a 12 h light-dark cycle and allowed free access to food and water before the experiment.

A total of 215 focal ischemic rats induced by middle cerebral artery occlusion (MCAO) were randomly assigned to different experimental groups. Group sizes of *n* = 8 were used. For the dose-ranging study, rats were divided into the vehicle group, maslinic acid-treated groups at 0.4, 2 and 10 μg/mL, and the vehicle group, MK-801-treated groups at 0.25, 0.5 and 1 mg/kg. In the time window study, rats were divided into 10 groups: the vehicle group, maslinic acid-treated group, MK-801–treated groups at 1 h, 2 h, 3 h and 4 h after ischemia and MK-801 combined with maslinic acid groups at 1h, 2 h, 3 h and 4 h after ischemia. To confirm the effective dosage of the combination therapy, rats were divided into the vehicle group, maslinic acid-treated group, MK-801-treated group and the combination group.

Transient MCAO was performed as previously described [26]. Rats were anesthetized with chloral hydrate (400 mg/kg i.p.). The right common carotid artery (CCA), external carotid artery (ECA) and internal carotid artery (ICA) were exposed via a ventral midline incision. A 3-0 monofilament nylon suture with a rounded tip was inserted into the ECA lumen and gently advanced to the ICA until slight resistance is felt. Relative cerebral blood flow (rCBF) was measured using a laser Doppler flowmeter (Moor Instruments, Axminster, UK). After thinning the skull −2.0 mm posterior and 4 mm lateral to bregma, the laser Doppler probe was affixed to the skull using α-cyanoacrylate adhesive. Blood flow dropped to <30% of the baseline and remained at the level throughout the occlusion period. After 2 h of occlusion, MCA blood flow was restored by withdrawal of the suture. The rectal temperature was monitored and kept at 37.0 ± 0.5 °C with a thermostatically controlled heating pad. Left femoral artery was catheterized for continuous arterial blood gas monitoring to make sure the physiological conditions were the same in vehicle- and treated animals at baseline. Sham-operated animals underwent the same surgical procedure, with the omission of MCAO.

### 4.3. Drug Administration

Animals were randomly assigned in a blinded fashion. Maslinic acid was dissolved in DMSO and then was diluted in artificial cerebrospinal fluid (aCSF) at the concentrations of 0.4 μg/mL, 2 μg/mL and 10 μg/mL, with a final DMSO concentration being 1%. The composition of the aCSF was as follows (in mM): 120 NaCl, 2.7 KCl, 1 MgSO_4_, 1.2 CaCl_2_, 25 NaHCO_3_ and 0.05 ascorbic acid (pH 7.3). Maslinic acid was administered intracerebroventricularly (i.c.v.) as previously described [27]. Rats were anesthetized with chloral hydrate and placed in a stereotaxic apparatus (Kopf Instruments, Tujunga, CA, USA). A volume of 5 μL maslinic acid or aCSF was injected into the right lateral cerebral ventricle (1.2 mm laterally, 0.84 mm posteriorly from bregma, depth 4.2 mm), using a microinjector 15 min before MCAO. MK-801 at 0.25 mg/kg, 0.5 mg/kg and 1 mg/kg was dissolved in saline and 0.5 mg/kg MK-801 was administered at different time point after ischemia induction by intravenous injection (i.v.). The protocol was shown in Figure 1B.

### 4.4. Measurement of Infarct Volume

The brain was removed and sectioned coronally into 2-mm-thick slices. Slices were stained with 2% TTC at 37 °C for 10 min, followed by 10% formalin for 1 h. The infarction areas on each slice were measured using ImageJ software. White or pink brain tissue was considered as ischemic region whereas red tissue was considered viable. The infarct volume was calculated by multiplying the ischemic area from each section by the slice thickness. The infarct volume was expressed as percentage of hemisphere volume and adjusted for edema [28]. 

### 4.5. Histology

Rats were sacrificed by deeply anesthetized with chloral hydrate and transcardially perfused with saline (200 mL) followed by 4% paraformaldehyde (200 mL). The brain was quickly removed and fixed in 4% paraformaldehyde at 4 °C for 4 h. The brain was then embedded in paraffin, and was cut into coronal sections of 4 μm thickness with a cryostat. Sections were rehydrated, followed by H&E staining for the assessment of neuronal damage in the CA1 region of the hippocampus. The sections were examined with a light microscope and the hippocampal neuronal damage was evaluated by using graded neuropathology score (0–4 point scale) as previously described [29]: grade 0, no damage to any hippocampal subregion; grade 1, scattered ischemic neurons in CA1 subregion; grade 2, moderate ischemic damage in CA1 subregion (less than half of pyramidal cells affected); grade 3, severe damage to pyramidal cells in CA1 subregion (more than half of pyramidal cells affected); grade 4, extensive cell damage in all hippocampal subregions. Quantitative assays of CA1 pyramidal neurons were performed for each group to examine the total loss of pyramidal neurons. Normal neurons were identified by their shape and size in the same designated area of the CA1 subregion [30]. Neuronal damage was evaluated by two individuals who were unaware of the treatment conditions. Data were obtained from three independent animals containing three randomly selected fields. 

### 4.6. Immunohistochemistry

Coronal sections were prepared as described above. Sections were washed and treated with 0.3% H_2_O_2_ for 10 min. Nonspecific binding sites were blocked with 4% goat serum. The sections were incubated overnight with primary antibody (GFAP, 1:600 dilution, GLAST, 1:500 dilution and GLT-1, 1:200 dilution). The primary antibodies were detected by DAKO EnVision-HRP for 30 minutes at room temperature and developed with DAB. Brain sections were analyzed with a light microscope and cells in each microscopic field were determined using Image-Pro Plus 6.0 software (Media Cybernetics, Bethesda, MD, USA). Dark brown dots were counted as positive cells [31,32]. Data were obtained from three independent animals containing three randomly selected fields.

### 4.7. Western Blot

Protein samples from hippocampus were homogenized using RIPA lysis buffer. Equal amounts of protein per sample were loaded in each lane and separated by SDS-PAGE, and transferred to nitrocellulose membranes in Tris-glycine buffer (48 mM Tris, 39 mM glycine, pH 9.2) containing 20% methanol. The membranes were blocked with 5% skimmed milk for 1 h, washed in Tris buffered saline containing 0.1% Tween-20 (TBST), and incubated with the primary antibodies overnight at 4 °C. After washing in TBST for three times, the nitrocellulose membranes were incubated for 1 h at room temperature with the horseradish peroxidase conjugated goat anti-rabbit or anti-mouse IgG. The protein bands detected by the antibodies were visualized using the SuperSignal West Pico Chemiluminescent Substrate Trial Kit (Pierce, Rockford, IL, USA). Images were taken using the ChemiDoc XRS system with Quantity One software (Bio-Rad, Richmond, CA, USA). The expression of target proteins were normalized to GAPDH expression.

### 4.8. Statistical Analysis

SPSS Statistics (SPSS Inc., Chicago, IL, USA) was used for the statistical analyses. All data were presented as means ± SEM of at least three independent preparations. Statistical analyses were performed by one-way ANOVA followed by a Tukey post-hoc test. The P value of less than 0.05 was considered statistically significant.

## 5. Conclusions

In summary, the results demonstrate that the synergistic effect provided by maslinic acid on the therapeutic time window and dosage range for MK-801 is probably attributed to the modulation of glutamate excitotoxicity by up-regulating the astrocytic glutamate transporter GLT-1 levels and promoting astrocyte activation. It can be stated that drugs targeting astrocytic glutamate transporters in combination with NMDA receptor antagonists may be a promising avenue for the future treatment of stroke.
